# Eating self-efficacy changes in individuals with type 2 diabetes following a structured lifestyle intervention based on the transcultural Diabetes Nutrition Algorithm (tDNA): A secondary analysis of a randomized controlled trial

**DOI:** 10.1371/journal.pone.0242487

**Published:** 2020-11-30

**Authors:** Harvinder Kaur Gilcharan Singh, Winnie Siew Swee Chee, Osama Hamdy, Jeffrey Ian Mechanick, Verna Kar Mun Lee, Ankur Barua, Siti Zubaidah Mohd Ali, Zanariah Hussein

**Affiliations:** 1 Division of Nutrition and Dietetics, School of Health Sciences, International Medical University, Kuala Lumpur, Malaysia; 2 Division of Endocrinology, Diabetes and Metabolism, Joslin Diabetes Center, Harvard Medical School, Boston, United States of America; 3 Division of Endocrinology, Diabetes and Bone Disease, Icahn School of Medicine at Mount Sinai, New York, United States of America; 4 Department of Family Medicine, School of Medicine, International Medical University, Kuala Lumpur, Malaysia; 5 Department of Community Medicine, School of Medicine, International Medical University, Kuala Lumpur, Malaysia; 6 Department of Non-Communicable Diseases, Klinik Kesihatan Seremban, Negeri Sembilan, Malaysia; 7 Department of Medicine, Hospital Putrajaya, Pusat Pentadbiran Kerajaan Persekutuan, Putrajaya, Malaysia; Weill Cornell Medical College in Qatar, QATAR

## Abstract

**Objective:**

Eating self-efficacy behavior is an important predictor of successful lifestyle intervention. This secondary analysis evaluated the changes in eating self-efficacy behavior in patients with type 2 diabetes (T2D) and overweight/obesity following structured lifestyle intervention based on the Malaysian customized transcultural Diabetes Nutrition Algorithm (tDNA).

**Methods:**

Patients with T2D and overweight/obesity (n = 230) were randomized either into the tDNA group which included a structured low-calorie meal plan using normal foods, incorporation of diabetes-specific meal replacements, and an exercise prescription or usual T2D care (UC) for 6 months. Patients in the tDNA group also received either counseling with motivational interviewing (tDNA-MI) or conventional counseling (tDNA-CC). The UC group received standard dietary and exercise advice using conventional counseling. Eating self-efficacy was assessed using a locally validated Weight Efficacy Lifestyle (WEL) questionnaire. All patients were followed up for additional 6 months’ post-intervention.

**Results:**

There was a significant change in WEL scores with intervention over one-year [Group X Time effect: F = 51.4, df = (3.4, 318.7), p<0.001]. Compared to baseline, WEL scores improved in both the tDNA groups with significantly higher improvement in the tDNA-MI group compared to the tDNA-CC and UC groups at 6 months (tDNA-MI: 25.4±2.1 vs. tDNA-CC: 12.9±2.8 vs. UC: -6.9±1.9, p<0.001). At 12 months’ follow-up, both the tDNA groups maintained improvement in the WEL scores, with significantly higher scores in the tDNA-MI group than tDNA-CC group, and the UC group had decreased WEL scores (tDNA-MI: 28.9±3.1 vs. tDNA-CC: 11.6±3.6 vs. UC: -13.2±2.1, p<0.001). Patients in the tDNA-MI group with greater weight loss and hemoglobin A1C reduction also had a higher eating self-efficacy, with a similar trend observed in comparative groups.

**Conclusion:**

Eating self-efficacy improved in patients with T2D and overweight/obesity who maintained their weight loss and glycemic control following a structured lifestyle intervention based on the Malaysian customized tDNA and the improvement was further enhanced with motivational interviewing.

**Clinical trial:**

This randomized clinical trial was registered under National Medical Research Registry, Ministry of Health Malaysia with registration number: NMRR-14-1042-19455 and also under ClinicalTrials.gov with registration number: NCT03881540.

## Introduction

The global increase in prevalence of type 2 diabetes mellitus (T2D) seen in the recent years is largely attributed to obesity, unhealthy dietary patterns, and physical inactivity. Malaysia has a rising prevalence of T2D affecting 17.5% of the adult population [1] with 83.4% of Malaysians with T2D being obese (75% of which have abdominal obesity) [2, 3]. Only 16.4% of patients with T2D in Malaysia adhere to a healthy eating pattern provided by dietitians [4], while 33.5% of Malaysian adults do not engage in the recommended amount of physical activity [1].

It is well established that Medical Nutrition Therapy (MNT), consisting of dietary and physical activity recommendations, is the cornerstone of optimal diabetes care [5]. To enable the broad implementation of these recommendations, the international task force of diabetes and nutrition experts developed the global trans-cultural diabetes nutrition algorithm (tDNA) to facilitate the delivery of nutrition therapy to patients with T2D and prediabetes in a variety of cultures, ethnic and geographic locations [6]. The global tDNA was later adapted for the Malaysian application in view of our unique disease characteristics, risk factors, cultural and lifestyle dissimilarities [7]. We recently published the effectiveness of a structured lifestyle intervention based on the tDNA, delivered using validated behavioral approaches such as the motivational interviewing (MI) and conventional counseling (CC) on weight and hemoglobin A1C (A1C) changes in patients with T2D and overweight/obesity [8]. Patients who received the tDNA intervention with MI counseling, when compared to patients receiving tDNA intervention with CC and usual diabetes care (UC) had significantly greater weight loss (tDNA-MI: -6.9 ± 1.3 kg vs. tDNA-CC: -5.3 ± 1.2 kg vs. UC: -0.8 ± 0.5 kg, p<0.001) and A1C reduction (tDNA-MI: -1.1 ± 0.1% vs. tDNA-CC: -0.5 ± 0.1% vs. UC: -0.2 ± 0.1%, p<0.001) after 6 months of intervention, and the benefits were maintained for additional 6 months [8]. In this secondary analysis, we aim to explore the effectiveness of the tDNA intervention on patients’ eating self-efficacy.

Eating self-efficacy is the confidence in an individual’s own ability to perform specific tasks to attain certain goals in challenging situations [9]. It is the key concept of Bandura’s social cognitive theory that behavior is strongly stimulated by self-influence [9]. Based on this theory, it is recognized that individuals’ have self‐motivating, self‐reflecting, creative and self‐steering possibilities, which enable them to have some control over their thoughts, feelings and actions [10]. In weight loss interventions, patients with higher baseline eating self-efficacy demonstrated greater weight loss with interventions [11, 12] and their eating self-efficacy improved after completion of a dedicated program [13–15].

Self-efficacy is overwhelmingly important to study as it is a malleable factor that predicts behavior change within multiple theories such as the social cognitive theory (Bandura, 1986) [16], goal-setting theory (Locke and Latham, 2002) [17], the health belief model (Rosenstock, Strecher, and Becker, 1988) [18], self-efficacy theory (Bandura, 1997) [19], the transtheoretical model (Prochaska and Velicer, 1997) [20], and the theory of planned behavior (in the form of ‘perceived behavioral control’; Ajzen, 1985) [21]. In weight loss interventions, self-efficacy plays an important role in overcoming barriers to behavior change [11, 22, 23]. Improved self‐efficacy may effectively contend with lifestyle barriers associated with improvements in weight management behaviors [11, 22, 23]. A recent systematic review and meta-analysis showed that self-efficacy-focused diabetes education successfully led to better psychosocial and metabolic outcomes in patients with T2D [23]. Besides being a known predictor of weight loss [11, 12, 24] and weight loss maintenance [13, 14, 25], eating self-efficacy is also an important predictor of the initiation and performance of weight control behaviors [24, 26]. Linde et al. [24], found that greater eating self-efficacy prospectively predicted weight-loss behaviors, such as higher total days in which individuals were adherent to the dietary plan, counted their calorie intake, and consumed less fat. Al-Khawaldeh et al. [26], reported better diet, exercise, blood glucose, and medication monitoring behaviors in patients with T2D exhibiting higher self-efficacy. This, in turn, led to higher diabetes treatment satisfaction and better glycemic control in these patients [26, 27]. The attractiveness of self‐efficacy is confirmed by numerous investigations in the field of health behavior. These show that self‐efficacy is an important predictor for a wide range of health behaviors, such as weight control, nutrition, use of alcohol, smoking and among others [28]. In Malaysia, the relationship between self‐efficacy and health behavior of patients with T2D was investigated by a limited number of studies [29–31].

In this study, we specifically evaluated patients’ self-efficacy to resist eating in challenging situations. This eating self-efficacy behavior was of particular interest because individuals with overweight/obesity tend to have low confidence to resist eating at high risk situations such as social gatherings, when in stress, during food availability and eating in front of television leading to poor weight outcomes [32]. This eating self-efficacy behavior is commonly measured using the Weight Efficacy Lifestyle (WEL) questionnaire, developed by Clark and colleagues [11]. Based on the WEL questionnaire, eating self-efficacy can be described as the confidence in resisting to eat in five tempting situations which includes negative emotions, availability, social pressure, physical discomfort, and positive activities [11]. The WEL questionnaire is a 20-item Likert scale which is easy to use, valid, and reliable as a measure of eating self-efficacy, with an internal consistency Cronbach’s-α of 0.7–0.9 (good reliability) in Caucasian [11] and African-American [33] populations. Recently, the WEL questionnaire was validated for use among the Malaysian patients with T2D, yielding an internal consistency Cronbach’s-α of 0.8, an indication of a good reliability measure [34]. A short-form of the WEL questionnaire (SF-WEL) consisting of 8-items is also available for use [35], and has an internal consistency Cronbach’s-α of 0.92 (good reliability) [36]. However, in this study, eating self-efficacy was evaluated using the original 20-item WEL questionnaire.

Successful translation of lifestyle intervention from clinical trials to real-world clinical settings, requires incorporation of behavioral factors and one behavior, is that a person’s “eating self-efficacy”. This secondary analysis presents the changes in eating self-efficacy behavior of patients with T2D and overweight/obesity who received structured lifestyle intervention based on the Malaysian customized transcultural Diabetes Nutrition Algorithm (tDNA) [8].

## Materials and methods

### Design, recruitment and settings

The full study design and methods have been previously described by Chee et al. [8]. The study was a randomized, open label, clinical trial of patients with T2D and overweight/obesity, conducted with 6 months’ intervention and 6 months’ passive follow-up. The recruitment process started in the mid of year 2015 and the study completed in year end of 2016. The study site was a primary care clinic in Seremban town, Negeri Sembilan. Patients, aged 30–65 years, with body mass index (BMI) of >23 kg/m^2^, and A1C >7% were recruited. Eligible patients were not treated with insulin and had optimized pharmacotherapy for T2D management, with no changes in pharmacotherapy within the past 3 months prior to the study. We excluded patients who were pregnant, nursing, or with a history of serious illness or diabetes-related complications. Written informed consent was obtained from all patients before enrollment. The study was commenced locally upon approval from the institutional review committee (IMU-R134/2014) and by the National Medical Research Registry, Ministry of Health Malaysia (NMRR-14-1042-19455). However, post-study completion, it was registered in the ClinicalTrials.gov database with a registration number of NCT03881540 as per the requirement for publication.

### Protocol overview

Patients were randomized to receive usual diabetes care (UC) or a structured lifestyle intervention based on the Malaysian tDNA [7]. Patients in the tDNA group were further randomized to receive either counseling with motivational interviewing (tDNA-MI) or conventional counseling (tDNA-CC) techniques to improve adherence to the lifestyle recommendations. Randomization was carried out by the investigator using the random allocation software [37].

Patients who were randomized to receive tDNA care underwent initial risk stratification based on the Malaysian tDNA algorithm [7]. A structured low calorie meal plan of 1200 kcal/day for female and 1500 kcal/day for male patients was prescribed using natural foods, with the incorporation of one or two servings of a diabetes-specific formula as meal replacements, (Glucerna SR, Abbott Nutrition, USA) as well as physical activity of ≥150 min/week to create a deficit in the calorie intake and aid weight loss. Education about lifestyle modification was provided using a tDNA toolkit, consisting of a flip chart on healthy eating, 14-day meal plans, and culturally adapted information on physical activity and exercise. Patients were further randomized to receive either motivational interviewing (tDNA-MI) or conventional counseling (tDNA-CC) to promote adherence to the lifestyle recommendations.

The MI counseling provided is a collaborative, patient-centered counseling style for eliciting behavior change by helping patients explore self-motivational statements towards positive behavior and resolve ambivalence by expressing empathy through reflective listening, avoiding argument and direct confrontation, developing discrepancy between patients’ goals or values and their current behavior, adjusting to patient resistance, and supporting self-efficacy and optimism [38]. The conventional counseling was counselor-driven rather than patient-driven focusing on empathetic listening, education, persuasion, and encouragement [8].

Patients randomized into UC group received care based on the Malaysian clinical practice guidelines for T2D [39]. A conventional low calorie diet plan of 1200 kcal/day for female and 1500 kcal/day for male patients was prescribed using normal foods, with standard diabetes support and lifestyle education to promote lifestyle change, calorie intake reduction, and aid weight loss. Patients were counseled by the dietitian based on individualized care using the similar conventional counseling technique to facilitate positive behavioral change toward weight loss. Patients receiving the UC were followed-up every 3 months throughout the one-year study period.

Patients receiving tDNA care were followed-up monthly during the initial 6 months of intervention and subsequently every 3 months during the passive follow-up for a period of 6 months.

### Measurements

Eating self-efficacy of patients was measured using WEL questionnaire validated in Malaysian patients with T2D [34]. Patients rated their self-efficacy on the 10-point Likert scale, where a score of 0 indicates “not confident” and a score of 9 indicates “very confident” to resist the desire to eat in tempting situations. There are five subscales of the WEL questionnaire, where each subscale is comprised of four items as shown in Table 1. The negative emotions subscale assesses the confidence to resist eating when feeling anxious, depressed, angry, or of failure. The availability subscale assesses a person's ability to resist poor eating habits on the weekends, when different foods are around, during a party, and knowing when certain high-calorie foods are available. The social pressure subscale focuses on declining food when others are encouraged to eat, when it is considered discourteous to refuse a second helping, when others are pressuring to eat, and when others may be upset. The physical discomfort subscale focuses on resistance to eat during times of bodily discomfort, fatigue, headache, or run down. Lastly, the positive activities subscale assesses resistance to eat when watching television, reading, preparing for bed, or being happy. The scores for each subscale ranges from 0–36 and a global sum of the subscales ranges from 0–180. Higher WEL scores indicate higher self-efficacy to resist eating.

**Table 1:** Weight Efficacy Lifestyle subscales and assessment items.

Subscales	Items
Negative emotions	I can resist eating when I am anxious (nervous)
	I can resist eating when I am depressed (or down)
	I can resist eating when I am angry (or irritable)
	I can resist eating when I have experienced failure
Availability	I can control my eating on weekends
	I can resist eating even when there are different kinds of food available
	I can resist eating even when I am at a party
	I can resist eating even when high calorie foods are available
Social pressure	I can resist eating when I have to say “no” to others
	I can resist eating even when I feel it's impolite to refuse a second helping
	I can resist eating even when others are pressuring me to eat
	I can resist eating even when I think others will be upset if I don’t eat
Physical discomfort	I can resist eating when I feel physically run down
	I can resist eating when I have a headache
	I can resist eating when I am in pain
	I can resist eating when I feel uncomfortable
Positive activities	I can resist eating when I am watching television
	I can resist eating when I am reading
	I can resist eating just before going to bed
	I can resist eating when I am happy

Patients’ weight was measured using a digital scale (Tanita HD-319, Tanita Corporation, Japan) and height was measured using a stadiometer (SECA, Hamburg, Germany). BMI was calculated. Blood was withdrawn from patients after an overnight fast of 10–12 hours and analyzed for A1C using automated procedures.

### Sample size

The sample size calculation for the main study was previously described in Chee et al. [8], where a total of 230 patients inclusive of 15% dropouts (115 patients receiving usual care; 115 patients receiving tDNA care) were sufficiently powered to examine the main study outcomes (weight and A1C). A post-hoc power analysis for this secondary objective, found that the existing sample size had power of greater than 70% at a significance level of 5%. These assumptions were tested to detect a significant difference of approximately 10% in absolute changes in total WEL scores from baseline to 6 and 12 months, between the three groups using analysis of covariance.

### Statistical analysis

Data was analyzed using the statistical package for social sciences (SPSS) version 25 software (Armonk, NY, US). This secondary analysis was carried out for study completers. The baseline differences between groups were analyzed using chi-square (χ^2^) test for categorical outcomes and one-way analysis of variance (ANOVA) or Kruskal-Wallis tests for continuous outcomes. A mixed-effects model for repeated measures was used to determine group-by-time interaction changes in WEL scores from baseline to 12 months. An unstructured covariance model for repeated measures was suitable for this analysis because 1) we assumed the observations on different study patients were independent which is legitimate for randomized designs; and 2) the covariance between observations at different times on the same patient are not the same. The outcomes were change scores calculated using 6- or 12- month scores minus baseline scores of WEL, weight and A1C. Because there was a significant between-group difference in baseline total WEL scores, sensitivity analysis was performed in which baseline total WEL scores was added as a covariate to the model while performing analysis of covariance (ANCOVA). Adding the covariate did not significantly alter the findings, hence, only the results adjusted for intervention groups from the repeated measures analysis were presented. Bonferroni adjustment for multiple comparisons overtime was reported. All p values of less than 0.05 were statistically significant.

## Results

### Baseline characteristics

The baseline characteristics of the study patients (n = 230) are summarized in Table 2. Patients in the tDNA-CC and UC groups had similar mean age of 53 ± 6 years and patients in the tDNA-MI group had a mean age of 54 ± 7 years. The majority of patients were female in the tDNA groups compared to the UC group (tDNA-MI 67%; tDNA-CC 84%; and UC 49%). All three groups had higher percentage of Indians (tDNA-MI 72.4%; tDNA-CC 63.2%; and UC 48.7%), followed by Chinese (tDNA-MI 22.4%; tDNA-CC 19.3%; and UC 34.8%) and Malay patients (tDNA-MI 5.2%; tDNA-CC 17.5%; and UC 16.5%). This is in line with high prevalence of T2D among Malaysian Indians in the country. More than 90% of the patients were married. The median diabetes duration was significantly (p = 0.047) lower in the tDNA-CC group (6 ± 3 years) than in tDNA-MI (8 ± 5 years) and UC (7 ± 5 years) groups. Hypertension and dyslipidaemia were the major co-morbid conditions, with no significant difference in the co-morbidities between groups (34.5% tDNA-MI, 35.1% tDNA-CC, and 35.7% UC). Patients with T2D and overweight/obesity recruited in this study had similar baseline weight, BMI, and A1C with no significant difference between groups. However, there was a significant difference in the baseline total WEL scores between the groups (tDNA-MI: 126.5 ± 15.0 vs. tDNA-CC: 130.0 ± 21.0 vs. UC: 124.0 ± 31.0; p = 0.004). Patients in the tDNA groups had significantly higher WEL scores for resisting eating when experiencing negative emotions (p = 0.014), physical discomfort (p<0.001) and positive activities (p = 0.002) compared to the UC group. No significant difference between groups was observed for WEL scores in resisting eating when food is available (p = 0.139) and when there is social pressure (p = 0.368).

**Table 2:** Baseline characteristics of the study patients (n = 230).

	tDNA (n = 115)			
	tDNA-MI (n = 58)	tDNA-CC (n = 57)	UC (n = 115)	p-value
Age (years)^a^	54 ± 7	53 ± 6	53 ± 6	0.953
Female (n, % patients)	39 (67)	48 (84)	56 (49)	<0.001*
Ethnicity (n, % patients)				0.014*
Malays	3 (5.2)	10 (17.5)	19 (16.5)	
Chinese	13 (22.4)	11 (19.3)	40 (34.8)	
Indians	42 (72.4)	36 (63.2)	56 (48.7)	
Duration of diabetes (years)	8 ± 5	6 ± 3	7 ± 5	0.047*
Weight (kg)	78.3 ± 20.9	74.6 ± 16.0	75.8 ± 18.6	0.218
Body mass index (kg/m^2^)	30.7 ± 8.2	29.4 ± 7.3	28.9 ± 6.3	0.171
A1C (%)	7.7 ± 1.1	7.7 ± 1.4	7.9 ± 1.3	0.368
A1C (mmol/mol)	61 ± 12	61 ± 15	63 ± 14	0.368
Weight Efficacy Lifestyle (WEL) scores				
Negative emotions	31.0 ± 4.3	31.0 ± 3.5	30.0 ± 4.0	0.014*
Availability	18.5 ± 8.3	20.0 ± 7.5	18.0 ± 10.0	0.139
Social pressure	24.0 ± 8.0	24.0 ± 9.5	24.0 ± 12	0.368
Physical discomfort	30.0 ± 4.0	30.0 ± 3.0	28.0 ± 5.0	<0.001*
Positive activities	26.0 ± 5.0	27.0 ± 5.0	24.0 ± 6.0	0.002*
Total WEL	126.5 ± 15.0	130.0 ± 21.0	124.0 ± 31.0	0.004*

Abbreviations: tDNA-MI; transcultural diabetes nutrition algorithm-motivational interviewing, tDNA-CC; transcultural diabetes nutrition algorithm—conventional counseling, A1C; hemoglobin A1C

^a^ Data expressed as mean ± standard deviation.

Otherwise data expressed as median ± interquartile range or frequency (percentage) patients.

*Significant differences between groups at p<0.05.

**Fig 1:**
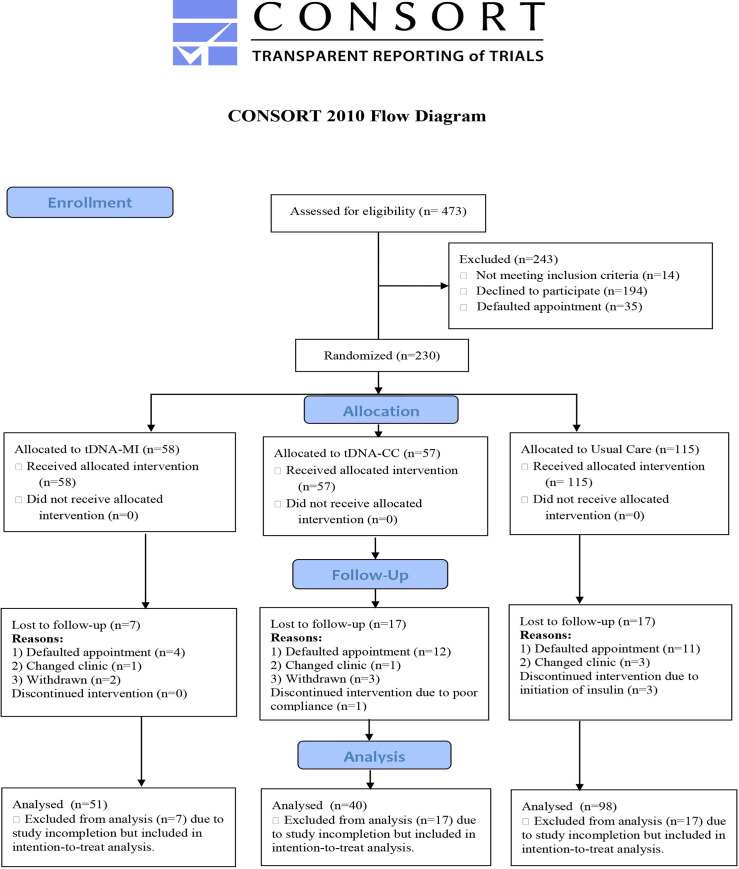
CONSORT flow diagram of patient retention rate.

A total of 230 patients with T2D and overweight/obesity participated in this study with only 189 completing the study, yielding an overall and an acceptable dropout rate of 17.8% as shown in Fig 1.

### Changes in WEL scores from baseline to 12 months of intervention

Comparing to baseline, at 6 months’ patients in both the tDNA groups had improvement in the eating self-efficacy scores, with a significantly greater increase in the total WEL scores observed for patients in the tDNA-MI group compared to patients in the tDNA-CC and UC groups (tDNA-MI: 25.4 ± 2.1 vs. tDNA-CC: 12.9 ± 2.8 vs. UC: -6.9 ± 1.9, p<0.001). A similar pattern of improvement was observed for all the WEL subscales such as negative emotions (tDNA-MI: 5.1 ± 0.6 vs. tDNA-CC: 2.9 ± 0.6 vs. UC: 1.5 ± 0.4, p<0.001), food availability (tDNA-MI: 7.6 ± 0.8 vs. tDNA-CC: 3.9 ± 1.1 vs. UC: -1.9 ± 0.7, p<0.001), social pressure (tDNA-MI: 4.1 ± 0.8 vs. tDNA-CC: 1.9 ± 1.0 vs. UC: -4.9 ± 0.7, p<0.001), physical discomfort (tDNA-MI: 3.2 ± 0.5 vs. tDNA-CC: 1.2 ± 0.5 vs. UC: -0.9 ± 0.4, p<0.001) and positive activities (tDNA-MI: 5.4 ± 0.6 vs. tDNA-CC: 3.2 ± 0.7 vs. UC: -0.8 ± 0.5, p<0.001) as shown in S1 Table. Pairwise multiple comparisons table and the change scores as shown in Table 3. The pairwise multiple comparisons at 6 months of intervention showed significant difference of scores between groups for the tDNA-MI vs. UC groups and tDNA-CC vs. UC groups but not for the tDNA-MI vs. tDNA-CC groups.

**Table 3:** Weight Efficacy Lifestyle (WEL) scores change overtime from baseline to 12 months of intervention (mean ± SE).

	tDNA (n = 91)			Group X Time interaction effect	
	tDNA-MI (n = 51)	tDNA-CC (n = 40)	UC (n = 98)	F(df)	p-values^a^
**Negative emotions scores**					
Baseline	29.5 ± 0.5	30.9 ± 0.5	29.1 ± 0.3		
Change at 6 months	5.1 ± 0.6	2.9 ± 0.6	1.5 ± 0.4		
Change at 12 months	5.3 ± 0.6	3.1 ± 0.6	-0.6 ± 0.5	23.5 (3.2, 297.5)	<0.001*
**Availability scores**					
Baseline	19.7 ± 0.8	21.3 ± 0.9	18.3 ± 0.6		
Change at 6 months	7.6 ± 0.8	3.9 ± 1.1	-1.9 ± 0.7		
Change at 12 months	9.3 ± 1.0	3.1 ± 1.4	-2.6 ± 0.7	33.8 (3.4, 317.5)	<0.001*
**Social pressure scores**					
Baseline	24.2 ± 0.8	24.8 ± 0.9	22.6 ± 0.6		
Change at 6 months	4.1 ± 0.8	1.9 ± 1.0	-4.9 ± 0.7		
Change at 12 months	4.3 ± 1.1	0.3 ± 1.3	-6.2 ± 0.7	28.9 (3.3, 309.3)	<0.001*
**Physical discomfort scores**					
Baseline	29.8 ± 0.5	30.7 ± 0.5	27.5 ± 0.3		
Change at 6 months	3.2 ± 0.5	1.2 ± 0.5	-0.9 ± 0.4		
Change at 12 months	3.8 ± 0.6	1.3 ± 0.8	-2.7 ± 0.5	23.1 (3.6, 339.0)	<0.001*
**Positive activities scores**					
Baseline	25.8 ± 0.6	27.1 ± 0.6	24.4 ± 0.4		
Change at 6 months	5.4 ± 0.6	3.2 ± 0.7	-0.8 ± 0.5		
Change at 12 months	6.2 ± 0.6	3.9 ± 0.6	-1.2 ± 0.5	31.6 (3.6, 334.4)	<0.001*
**Total WEL scores**					
Baseline	129.1 ± 2.3	134.7 ± 2.6	121.9 ± 1.6		
Change at 6 months	25.4 ± 2.1	12.9 ± 2.8	-6.9 ± 1.9		
Change at 12 months	28.9 ± 3.1	11.6 ± 3.6	-13.2 ± 2.1	51.4 (3.4, 318.7)	<0.001*

Abbreviations: tDNA-MI; transcultural diabetes nutrition algorithm-motivational interviewing, tDNA-CC; transcultural diabetes nutrition algorithm-conventional counseling, WEL; Weight Efficacy Lifestyle

All data expressed as (mean ± SE) for absolute change in measures.

Data presented for completers at 6 months are based on total sample of n = 192 (tDNA-MI = 51, tDNA-CC = 40 and UC = 101).

^a^p-values measures group-by-time interaction by mixed-effects model repeated measures test adjusted for intervention group with Bonferroni adjustments for multiple comparisons.

*Significant changes from baseline at p<0.05.

Table 3 shows the group-by-time interaction effect of change in eating self-efficacy scores from baseline to 12 months of intervention. There was a significant change in eating self-efficacy scores overtime with the intervention provided in patients with T2D and overweight/obesity when experiencing negative emotions [Group X Time effect: F = 23.5, df = (3.2, 297.5), p<0.001], during food availability [Group X Time effect: F = 33.8, df = (3.4, 317.5), p<0.001], when experiencing social pressure [Group X Time effect: F = 28.9, df = (3.3, 309.3), p<0.001], when in physical discomfort [Group X Time effect: F = 23.1, df = (3.6, 339.0), p<0.001], during positive activities [Group X Time effect: F = 31.6, df = (3.6, 334.4), p<0.001] and for total WEL [Group X Time effect: F = 51.4, df = (3.4, 318.7), p<0.001].

The changes in WEL scores at 1-year post-intervention is also shown in Table 3 and the pairwise comparisons is shown in S1 Table. Pairwise multiple comparisons table. There was a significant difference between groups, where patients in the tDNA-MI group continued to demonstrate a greater improvement of scores in all the WEL subscales and total WEL, while minimal improvement or no change in scores were observed for patients in the tDNA-CC group and the eating self-efficacy scores decreased for patients in the UC group especially when experiencing negative emotions (tDNA-MI: 5.3 ± 0.6 vs. tDNA-CC: 3.1 ± 0.6 vs. UC: -0.6 ± 0.5, p<0.001), during food availability (tDNA-MI: 9.3 ± 1.0 vs. tDNA-CC: 3.1 ± 1.4 vs. UC: -2.6 ± 0.7, p<0.001), when experiencing social pressure (tDNA-MI: 4.3 ± 1.1 vs. tDNA-CC: 0.3 ± 1.3 vs. UC: -6.2 ± 0.7, p<0.001), when in physical discomfort (tDNA-MI: 3.8 ± 0.6 vs. tDNA-CC: 1.3 ± 0.8 vs. UC: -2.7 ± 0.5, p<0.001), during positive activities (tDNA-MI: 6.2 ± 0.6 vs. tDNA-CC: 3.9 ± 0.6 vs. UC: -1.2 ± 0.5, p<0.001) and in terms of total WEL (tDNA-MI: 28.9 ± 3.1 vs. tDNA-CC: 11.6 ± 3.6 vs. UC: -13.2 ± 2.1, p<0.001). Similarly, at 12 months the pairwise multiple comparisons of significant difference in scores between groups were observed for the tDNA-MI vs. UC groups and tDNA-CC vs. UC groups but not for the tDNA-MI vs. tDNA-CC groups (except for food availability).

**Fig 2:**
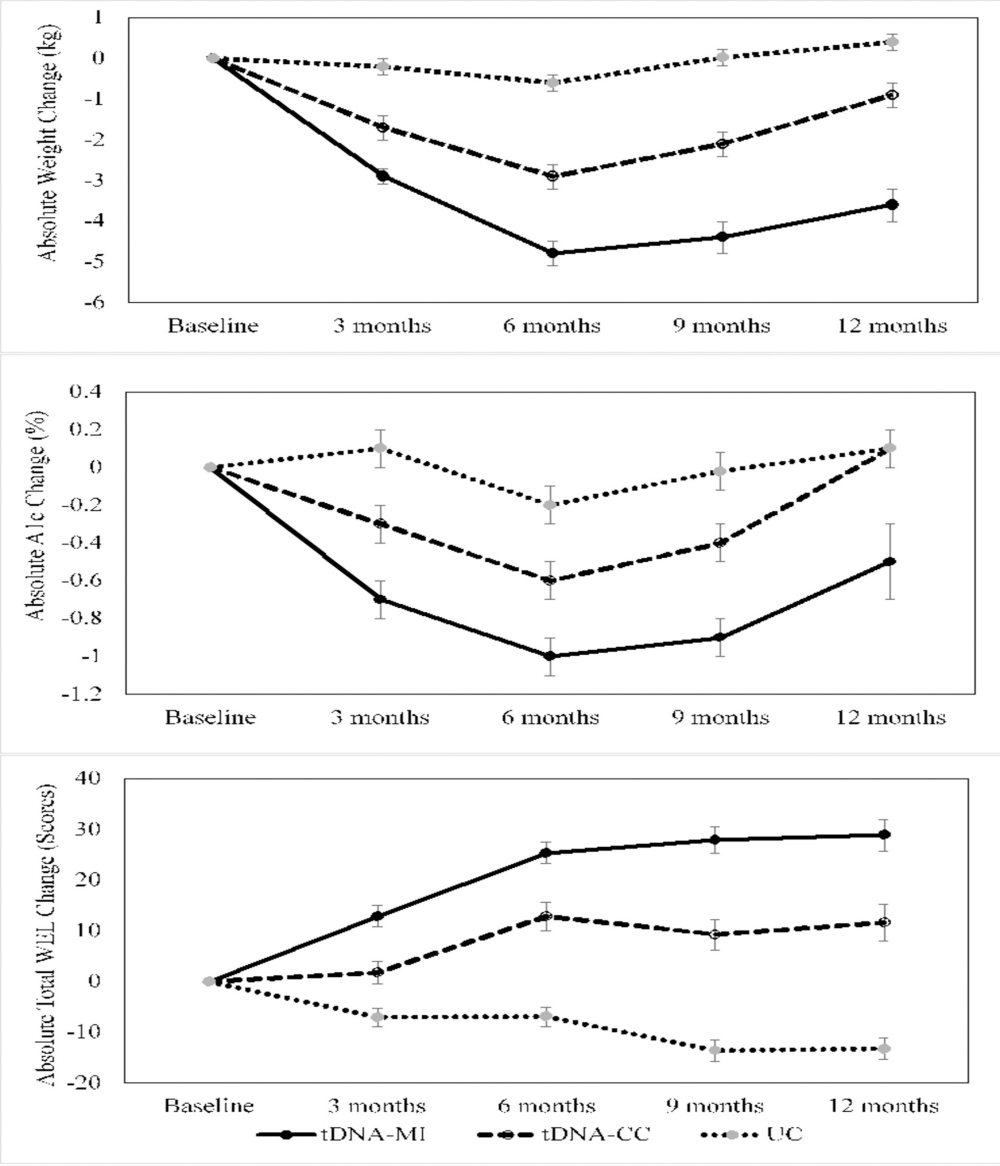
Illustration of absolute change in weight, glycated hemoglobin (A1C) and total Weight Efficacy Lifestyle (WEL) scores over time. Error bars shows between-group significance over time at p<0.05.

In view of between-group baseline differences, sensitivity analysis was performed and the results are shown in S2 Table. Sensitivity analysis table. After adjusting for baseline WEL scores, the findings did not alter the results of this study. Significant absolute change in WEL scores from baseline to 12 months in this secondary analysis remained the same as in Table 3. Results adjusted for intervention groups were presented in Table 3 and Fig 2.

### WEL scores and changes in weight and A1C

Fig 2 illustrates the changes in weight, A1C and total WEL scores overtime for study completers. Patients in the tDNA-MI group compared to patients in the tDNA-CC and UC groups, who had significantly greater weight loss (tDNA-MI: -4.8 ± 0.3 kg vs. tDNA-CC: -2.9 ± 0.3 kg vs. UC: -0.6 ± 0.2 kg, p<0.001) and A1C reduction (tDNA-MI: -1.0 ± 0.1% vs. tDNA-CC: -0.6 ± 0.1% vs. UC: -0.2 ± 0.1%, p<0.001), also significantly improved their eating self-efficacy (tDNA-MI: 25.4 ± 2.1 vs. tDNA-CC: 12.9 ± 2.8 vs. UC: -6.9 ± 1.9, p<0.001) over the 6 months’ period. Additionally, patients in the tDNA-MI group who maintained a significant weight loss (tDNA-MI: -3.6 ± 0.4 kg vs. tDNA-CC: -0.9 ± 0.3 kg vs. UC: 0.4 ± 0.2 kg, p<0.001) and A1C reduction (tDNA-MI: -0.5 ± 0.2% vs. tDNA-CC: 0.1 ± 0.1% vs. UC: 0.1± 0.1%, p = 0.007) after 1-year of trial completion, continued to maintain a significant improvement in their self-efficacy to resist eating in challenging situations (tDNA-MI: 28.9 ± 3.1 vs. tDNA-CC: 11.6 ± 3.6 vs. UC: -13.2 ± 2.1, p<0.001).

## Discussion

This secondary analysis demonstrated that eating self-efficacy as measured using WEL questionnaire improved in patients following a structured lifestyle intervention based on the Malaysian customized tDNA. We also demonstrated a correspondence between WEL scores with both weight loss and A1C reduction. Patients who had greater weight loss and A1C reduction also had higher total WEL scores and these results were consistent with other self-efficacy studies that showed improvements over the course of weight loss interventions and also predicted greater weight loss upon completion of the trial [15, 25, 33, 40–44]. Structured lifestyle intervention in other studies has also successfully improved patients’ self-efficacy level parallel to greater weight loss and better glycemic control [42–46]. Schillinger and colleagues [46], found that structured lifestyle intervention, when specifically applied to weight loss interventions, does not only increases patients’ self-efficacy level, but also improves patients’ satisfaction, knowledge, and coping behaviors [46].

In addition, this study showed that structured lifestyle intervention delivered with behavioral counseling techniques such as motivational interviewing can affect positively on the ability to resist eating in persuasive situations as measured by the subscales in the WEL questionnaire. Motivational interviewing is a patient-centered counseling approach, with the goal of eliciting patients’ self-motivational statements that results in greater intrinsic motivation to change [38]. Mirkarimi et al. [47], found that MI increases efficacy of lifestyle change on weight loss in patients with overweight/obesity with a more durable effect, compared to those receiving the nutrition education program in the control group. Evidence supports the positive impact of MI on patients’ self-efficacy level by improving patients’ psychological and physiological well-being towards positive behavior change [48–50]. Di Marco et al. [49], showed that MI not only increases perceived support, but also enhances commitment behaviors using increased self-efficacy and controlled eating in persuasive situations.

It is interesting to note that there were differences in scores in the negative emotions, physical discomfort, and positive activities subscales between groups at baseline in this study. Patients with obesity often experience more negative emotions, lose control of their food intake, and revert to overconsumption [51]. Difficulty in coping with negative emotions has been associated with reduced dietary compliance in patients with obesity [51, 52]. Furthermore, patients with obesity have poor coping mechanisms when in pain or any physical discomfort where they tend to overeat [53] and consequently revert to consuming foods high in calories, fat, and sugar to feel better [54, 55]. When engaging in positive activities, such as watching television or reading, food consumption can increase [55]. Past research indicates that television viewing, computer time, reading, music/radio listening, and other relaxation activities were associated with increased unhealthy food consumption and obesity [55, 56]. Nevertheless, when adjusted for baseline WEL scores as shown in the sensitivity analysis (S2 Table. Sensitivity analysis table), the results obtained did not alter this study finding. In this study, presented results in Table 3 and Fig 2 were adjusted for intervention groups.

It was also observed that there were higher percentage of female patients, Indians and patients with considerable long duration of diabetes in the tDNA groups than in UC group which corresponds with a higher eating self-efficacy level among the tDNA groups than the UC group. Our findings could be explained as numerous investigations showed female patients with overweight/obesity tend to lose more weight in weight management programs, thus, tend to have a higher self-efficacy than their male counterparts [25, 57]. On the contrary, there are also investigations reporting male patients with overweight/obesity tend to have better initial self-efficacy level in weight loss programs [58, 59] and another study among the Malaysian T2D population showed self-efficacy level was similar between genders [60]. Most investigations showed no ethnicity differences in eating self-efficacy [29, 61]. Additionally, Wu et al. reported that patients with longer history of T2D have higher self-efficacy [62] and improved self-efficacy in the diabetes self-care management [60].

Certain limitations of this study must be acknowledged. Firstly, there were significant differences in gender, ethnicity, duration of diabetes, negative emotions, physical discomfort, and total WEL scores between groups at baseline. However, when adjusted for baseline WEL scores, the sensitivity analysis showed the findings of this study was not significantly altered. The results presented were adjusted for intervention groups. Secondly, patients’ eating self-efficacy was evaluated using the survey-based WEL questionnaire, which can have a high response bias. Nevertheless, all interviews with the study patients were conducted face-to-face by the investigator to limit this bias. In addition, this study was conducted among patients with T2D and overweight/obesity who were on oral anti-diabetic agents and had lower severity of diabetes-related complications. This study finding is limited in its generalizability in patients with T2D on insulin pharmacotherapy and have greater severity of diabetes-related complications. This was a secondary analysis of an exploratory outcome which is the eating self-efficacy that had acceptable power analysis even though the study sample size was small. The small sample size limited the performance of sophisticated statistical modelling. Despite these limitations, this study is novel and demonstrates the importance of investigating eating self-efficacy of patients participating in weight loss interventions. Strengths of the current study are the randomized control study design, the relative long term follow-up of patients with T2D and overweight/obesity, the low attrition rate (17.8%) and the consideration of psychological as well as physiological variables.

## Conclusion

Our findings suggest that eating self-efficacy is an important behavioral component to be assessed in obesity-related interventions. In this study, eating self-efficacy improved in patients with T2D and overweight/obesity who maintained their weight loss and glycemic control following a structured lifestyle intervention based on the Malaysian customized tDNA and the improvement was further enhanced with motivational interviewing.

## Supporting information

S1 Checklist CONSORT checklist. (DOC)

S1 Table Pairwise multiple comparisons table. (DOCX)

S2 Table Sensitivity analysis table. (DOCX)

S1 File Study protocol. (DOCX)
