# Supplementary material for: Eating self-efficacy changes in individuals with type 2 diabetes following a structured lifestyle intervention based on the transcultural Diabetes Nutrition Algorithm (tDNA): A secondary analysis of a randomized controlled trial
Source: PLoS One. 2020 Nov 30;15(11):e0242487. doi: 10.1371/journal.pone.0242487 (PMC7703935; doi:10.1371/journal.pone.0242487)
Supplement: S1 Table — (DOCX) [file pone.0242487.s002.docx]

**S1 Table. Pairwise multiple comparisons table.**

Weight Efficacy Lifestyle (WEL) scores from baseline to 6 and 12 months of intervention (mean ± SE) for pairwise multiple comparisons between groups

|  | tDNA (n=91) | |  |  |  |  |  |
| --- | --- | --- | --- | --- | --- | --- | --- |
|  | tDNA-MI (n=51) | tDNA-CC (n=40) | UC  (n=98) | p-values^a^  (Group x Time interaction from baseline to 6 months and baseline to 12 months) | p-values^b^ (pairwise comparison between tDNA-MI and tDNA-CC) | p-values^c^  (pairwise comparisons between tDNA-MI and UC) | p-values^d^  (pairwise comparisons between tDNA-CC and UC) |
|  | **Negative emotions scores** | | |  |  |  |  |
| Baseline | 29.5 ± 0.5 | 30.9 ± 0.5 | 29.1 ± 0.3 |  |  |  |  |
| 6 months | 34.5 ± 0.2 | 33.8 ± 0.3 | 30.5 ± 0.2 | <0.001* | 1.000 | <0.001* | <0.001* |
| 12 months | 34.7 ± 0.3 | 34.0 ± 0.3 | 28.5 ± 0.3 | <0.001* | 1.000 | <0.001* | <0.001* |
|  | **Availability scores** | | |  |  |  |  |
| Baseline | 19.7 ± 0.8 | 21.3 ± 0.9 | 18.3 ± 0.6 |  |  |  |  |
| 6 months | 27.4 ± 0.5 | 25.2 ± 0.6 | 16.7 ± 0.4 | <0.001* | 1.000 | <0.001* | <0.001* |
| 12 months | 29.0 ± 0.8 | 24.4 ± 0.9 | 15.8 ± 0.5 | <0.001* | 0.045* | <0.001* | <0.001* |
|  | **Social pressure scores** | | |  |  |  |  |
| Baseline | 24.2 ± 0.8 | 24.8 ± 0.9 | 22.6 ± 0.6 |  |  |  |  |
| 6 months | 28.4 ± 0.6 | 26.6 ± 0.6 | 17.7 ± 0.3 | <0.001* | 1.000 | <0.001* | <0.001* |
| 12 months | 28.5 ± 0.9 | 25.1 ± 0.7 | 16.4 ± 0.4 | <0.001* | 0.067 | <0.001* | <0.001* |
|  | **Physical discomfort scores** | | |  |  |  |  |
| Baseline | 29.8 ± 0.5 | 30.7 ± 0.5 | 27.5 ± 0.3 |  |  |  |  |
| 6 months | 33.0 ± 0.3 | 31.8 ± 0.4 | 26.5 ± 0.3 | <0.001* | 1.000 | <0.001* | <0.001* |
| 12 months | 33.6 ± 0.4 | 31.9 ± 0.6 | 24.8 ± 0.3 | <0.001* | 0.265 | <0.001* | <0.001* |
|  | **Positive activities scores** | | |  |  |  |  |
| Baseline | 25.8 ± 0.6 | 27.1 ± 0.6 | 24.4 ± 0.4 |  |  |  |  |
| 6 months | 31.2 ± 0.3 | 30.2 ± 0.4 | 23.7 ± 0.3 | <0.001* | 1.000 | <0.001* | <0.001* |
| 12 months | 32.0 ± 0.4 | 30.9 ± 0.5 | 23.1 ± 0.3 | <0.001* | 1.000 | <0.001* | <0.001* |
|  | **Total WEL scores** | | |  |  |  |  |
| Baseline | 129.1 ± 1.9 | 134.7 ± 2.6 | 121.9 ± 1.6 |  |  |  |  |
| 6 months | 154.5 ± 1.3 | 147.6 ± 1.9 | 115.1 ± 1.3 | <0.001* | 1.000 | <0.001* | <0.001* |
| 12 months | 157.9 ± 2.5 | 146.3 ± 2.5 | 108.7 ± 1.4 | <0.001* | 0.135 | <0.001* | <0.001* |

Abbreviations: tDNA-MI; transcultural diabetes nutrition algorithm-motivational interviewing, tDNA-CC; transcultural diabetes nutrition algorithm-conventional counseling, WEL; Weight Efficacy Lifestyle

All data expressed as (mean ± SE) of scores at baseline, 6- and 12 months.

Data presented for completers at 6 months are based on total sample of n=192 (tDNA-MI=51, tDNA-CC=40 and UC=101).

^a^p-values measures group-by-time interaction by mixed-effects model repeated measures test adjusted for intervention groups with Bonferroni adjustments for multiple comparisons.

^b^p-values measures pairwise comparisons of change in scores between the tDNA-MI and tDNA-CC groups with Bonferroni adjustments for multiple comparisons.

^c^p-values measures pairwise comparisons of change in scores between the tDNA-MI and UC groups with Bonferroni adjustments for multiple comparisons.

^d^p-values measures pairwise comparisons of change in scores between the tDNA-CC and UC groups with Bonferroni adjustments for multiple comparisons.

^*^Significant changes from baseline at p<0.05.
