# Supplementary material for: Eating self-efficacy changes in individuals with type 2 diabetes following a structured lifestyle intervention based on the transcultural Diabetes Nutrition Algorithm (tDNA): A secondary analysis of a randomized controlled trial
Source: PLoS One. 2020 Nov 30;15(11):e0242487. doi: 10.1371/journal.pone.0242487 (PMC7703935; doi:10.1371/journal.pone.0242487)
Supplement: S2 Table — (DOCX) [file pone.0242487.s003.docx]

**S2 Table. Sensitivity analysis table.**

Weight Efficacy Lifestyle (WEL) scores change overtime from baseline to 6 and 12 months of intervention (mean ± SE) after adjusting for baseline WEL scores

|  | tDNA (n=91) | |  |  |
| --- | --- | --- | --- | --- |
|  | tDNA-MI (n=51) | tDNA-CC (n=40) | UC  (n=98) | p-values^a^ |
|  | **Negative emotions scores** | | |  |
| Baseline | 29.5 ± 0.5 | 30.9 ± 0.5 | 29.1 ± 0.3 |  |
| Change at 6 months | 5.1 ± 0.6 | 2.9 ± 0.6 | 1.5 ± 0.4 | <0.001* |
| Change at 12 months | 5.3 ± 0.6 | 3.1 ± 0.6 | -0.6 ± 0.5 | <0.001* |
|  | **Availability scores** | | |  |
| Baseline | 19.7 ± 0.8 | 21.3 ± 0.9 | 18.3 ± 0.6 |  |
| Change at 6 months | 7.6 ± 0.8 | 3.9 ± 1.1 | -1.9 ± 0.7 | <0.001* |
| Change at 12 months | 9.3 ± 1.0 | 3.1 ± 1.4 | -2.6 ± 0.7 | <0.001* |
|  | **Social pressure scores** | | |  |
| Baseline | 24.2 ± 0.8 | 24.8 ± 0.9 | 22.6 ± 0.6 |  |
| Change at 6 months | 4.1 ± 0.8 | 1.9 ± 1.0 | -4.9 ± 0.7 | <0.001* |
| Change at 12 months | 4.3 ± 1.1 | 0.3 ± 1.3 | -6.2 ± 0.7 | <0.001* |
|  | **Physical discomfort scores** | | |  |
| Baseline | 29.8 ± 0.5 | 30.7 ± 0.5 | 27.5 ± 0.3 |  |
| Change at 6 months | 3.2 ± 0.5 | 1.2 ± 0.5 | -0.9 ± 0.4 | <0.001* |
| Change at 12 months | 3.8 ± 0.6 | 1.3 ± 0.8 | -2.7 ± 0.5 | <0.001* |
|  | **Positive activities scores** | | |  |
| Baseline | 25.8 ± 0.6 | 27.1 ± 0.6 | 24.4 ± 0.4 |  |
| Change at 6 months | 5.4 ± 0.6 | 3.2 ± 0.7 | -0.8 ± 0.5 | <0.001* |
| Change at 12 months | 6.2 ± 0.6 | 3.9 ± 0.6 | -1.2 ± 0.5 | <0.001* |
|  | **Total WEL scores** | | |  |
| Baseline | 129.1 ± 2.3 | 134.7 ± 2.6 | 121.9 ± 1.6 |  |
| Change at 6 months | 25.4 ± 2.1 | 12.9 ± 2.8 | -6.9 ± 1.9 | <0.001* |
| Change at 12 months | 28.9 ± 3.1 | 11.6 ± 3.6 | -13.2 ± 2.1 | <0.001* |

Abbreviations: tDNA-MI; transcultural diabetes nutrition algorithm-motivational interviewing, tDNA-CC; transcultural diabetes nutrition algorithm-conventional counseling, WEL; Weight Efficacy Lifestyle

Data presented for completers at 6 months are based on total sample of n=192 (tDNA-MI=51, tDNA-CC=40 and UC=101).

All data expressed as (mean ± SE) for absolute change in measures with Bonferroni adjustments for multiple comparisons.

^a^p-values measures differences in scores between groups by analysis of covariance adjusted for baseline WEL scores.

^*^Significant changes from baseline at p<0.05.
