# Supplementary material for: Eating self-efficacy changes in individuals with type 2 diabetes following a structured lifestyle intervention based on the transcultural Diabetes Nutrition Algorithm (tDNA): A secondary analysis of a randomized controlled trial
Source: PLoS One. 2020 Nov 30;15(11):e0242487. doi: 10.1371/journal.pone.0242487 (PMC7703935; doi:10.1371/journal.pone.0242487)
Supplement: S1 File — (DOCX) [file pone.0242487.s004.docx]

**Study Title**

The Efficacy of a Transcultural Diabetes-Specific Nutrition Algorithm (tDNA) Care on Weight Loss and Metabolic Outcomes in Patients with Overweight, Obesity and Type 2 Diabetes Mellitus in an Outpatient Clinic Setting

**Participating Investigators:**

1. Dr. Winnie Chee Siew Swee

Designation: Professor, Nutrition & Dietetics, IMU

1. Dr Zanariah Hussein

Designation: Consultant Endocrinologist, Hospital Putrajaya

1. Dr Verna Lee Kar Mun

Designation: Family Medicine Specialist & Head of IMU Medical Centre

1. Dr Siti Zubaidah bt Mohd Ali

Designation: Family Medicine Specialist, Klinik Kesihatan Seremban

1. Osama Hamdy

Designation. Lecturer, Joslin Diabetes Center, Harvard Medical School, Boston, USA

1. Jeffery Ian Mechanick

Designation: Lecturer, Icahn School of Medicine at Mount Sinai, New York, USA

1. Dr Ankur Barua

Designation: Senior lecturer, Community Medicine, IMU

**Postgraduate candidate:**

Ms Harvinder Kaur a/p Gilcharan Singh

MSc Medical and Health Sciences, IMU

**1.0 Study objective**

To investigate the effectiveness of structured lifestyle intervention following a transcultural diabetes nutrition algorithm (tDNA) care compared to usual diabetes care in patients with overweight, obesity and type 2 diabetes (T2D), receiving either the motivational interviewing counseling or conventional counseling technique in an outpatient clinic setting.

**1.1 Specific Objectives**

1. To compare changes in the primary outcomes (weight, body mass index and glycated hemoglobin) in patients receiving structured lifestyle intervention based on tDNA care compared to usual diabetes care in addition to receiving either the motivational interviewing counseling or conventional counseling techniques at baseline, 6 months and 12 months of intervention.
2. To compare changes in the secondary outcomes (waist circumference, percentage body fat, fasting plasma glucose, lipid profile, high sensitivity C-Reactive Protein and blood pressure) in patients receiving structured lifestyle intervention based on tDNA care compared to usual diabetes care in addition to receiving either the motivational interviewing counseling or conventional counseling techniques at baseline, and 6 months of intervention.
3. To compare the changes in dietary intake, Weight Efficacy Lifestyle, exercise and physical activity in patients receiving structured lifestyle intervention based on tDNA care compared to usual diabetes care in addition to receiving either the motivational interviewing counseling or conventional counseling techniques at baseline, 6 months and 12 months of intervention.

**1.2 Research hypothesis**

Weight loss and glycated hemoglobin (A1C) level will be improved in patients following the tDNA care compared to usual diabetes care and the improvements will be greater in those receiving motivational interviewing counseling than conventional counseling.

**2.0 Introduction and literature review**

The prevalence of T2D in Malaysia among adults aged >18 years has escalated from 11.6% to 17.5% in 10 years, according to the latest National Health and Morbidity survey, (2015) [1,2]. The glycemic control continued to deteriorate where the mean A1C increased from 8.0% in 2003 to 8.66% in 2008, and 8.5% in 2013 [3, 4, 5]. Furthermore, only 22% of T2D patients achieved the glycemic target of A1C <7.0% in 2008, the lowest rate since 1998 [3, 4].

The increasing incidences of T2D have resulted in the parallel increase in the prevalence of overweight and obesity among Malaysians. According to the latest National Health and Morbidity survey, (2015), the prevalence of overweight among adults aged >18 years has increased from 29.1% in 2006 to 30.0% in 2013 while the prevalence of obesity increased from 14.0% in 2006 to 17.7% in 2013 [1, 6]. The National Diabetes Registry 2009-2012 showed, 83.4% of Malaysian T2D patients were obese [7], and abdominal obesity was prevalent in 75% of these patients [8].

Overweight/obesity not only increases the risk of developing T2D, but also complicates its management. Overweight/obesity increases insulin resistance and glucose intolerance, making diabetes in overweight/obese patients more difficult to treat pharmacologically [9, 10]. A review with meta-analysis showed that weight loss of at least 5-10% in overweight/obese T2D patients confers significant benefits in terms of decreasing insulin resistance, substantially improving glycemic control, reducing lipemia, and reducing blood pressure [11]. In this review, weight management appears to be an important therapeutic task for individuals with T2D [11]. Furthermore, in a 12-year observational study in the US, a history of intentional weight loss among T2D patients was associated with a 25% reduction in total mortality and intentional weight loss of 20-29 pounds (~9-13 kg) produced the largest reduction in mortality (33%) [12]. This study suggested that a period of intentional weight loss in T2D patients can have a positive effect on health many years later.

Considerable research has been done to evaluate the effectiveness of various intervention programs aimed at preventing and treating overweight/obesity in T2D patients [13-17]. These interventions include calorie or nutrient restrictions and diet modifications [13, 14], programming to promote physical activity for weight loss [15], combined diet and exercise interventions [16], as well as various behavioral programs aimed at lifestyle changes [17]. Healthy lifestyle changes are the cornerstones of prevention as well as management of T2D. Most successful lifestyle intervention programs in T2D have combined dietary restrictions, exercises and behavior modifications for weight loss [18, 19]. A randomized control trial conducted to determine the feasibility and effects of lifestyle changes designed to prevent or delay the onset of T2D found that, lifestyle changes can reduce the risk of progression to diabetes by 58% over four years in both men and women with impaired glucose tolerance [20]. A recent meta-analysis that studied the effect of lifestyle intervention in patients with T2D found a significant reduction in body mass index (BMI) (-0.29 kg/m²; p=0.014), A1C (-0.37%; p=0.001), systolic blood pressure (-0.16 mmHg: p=0.016) and diastolic blood pressure (-0.27 mmHg, p<0.001) compared to controls [21]. This meta-analysis concluded that lifestyle intervention, inclusive of change in diet, exercise and education showed significant benefits on glycemic control and cardiovascular risk factors in T2D patients.

Healthy lifestyle evidenced-based recommendations can be found in clinical practice guidelines and medical nutrition therapy (MNT) guidelines. Recommendations must be reconstructed to account for cultural differences in lifestyle, food availability and genetic factors to enable the broad implementation of these guidelines. In 2012, members of a task force in the United States developed an algorithm for the nutritional management of people with T2D and prediabetes [22]. Subsequently, algorithm nodes were populated with transcultural attributes to guide decisions. The resultant transcultural diabetes nutrition algorithm (tDNA) was simplified and optimized for global implementation and validation according to current standards for clinical practice guidelines development and cultural adaptation [22]. In view of worsening glycemic control among Malaysians and the unique cultural and lifestyle dissimilarities, a task force of diabetes and nutrition experts restructured the international guidelines to develop the Malaysian application of the tDNA [23]. The tDNA functions as a tool to facilitate the delivery of nutrition therapy to patients with T2D and prediabetes in a variety of cultures and geographic locations. The Malaysian tDNA care has recommended the use of diabetes-specific nutritional formulas as meal replacements (MRs) in overweight/obese T2D patients as part of the lifestyle management [23].

Meal replacement products fortified with vitamins and minerals and contain known energy and nutrition content, have been a popular choice for weight loss strategies [24-26]. Meal replacements have been shown to help calorie control [27] and the consumption of MRs conferred benefits in terms of increasing dietary intake of essential nutrients among dieters [28]. While the traditional very low-calorie diet interventions may cause subtle deficits of vitamins and/or other nutrients and may aggravate a borderline insufficiency state among dieters, the MRs intake are currently regarded as safe and effective method for weight loss [17, 24]. The MRs intake has been reported to be effective in initial weight loss [17] and weight loss maintenance [29, 30, 31] in overweight/obese T2D patients. In the Look AHEAD study, one-year results showed MRs used as part of comprehensive behavior and lifestyle intervention led to 8.6% weight reduction compared to 0.7% weight reduction in the control group which received standard diabetes support and education [17]. After four-years, the Look AHEAD trial reported that those who received the MRs as part of the intensive lifestyle management maintained a 4.7% weight loss of initial weight compared to 1.1% weight loss in the control group [29]. Further, several longer-term maintenance studies suggested that replacing one meal (or one meal and one snack) per day with meal/snack replacements was associated with superior weight loss maintenance [30, 31]. When MRs intake was compared to traditional diet plans, evidence showed that weight loss and glycemic control in the MR groups was greater than the traditional diet plan groups [32, 33]. A meta and pooling-analysis reported that obese individuals receiving MRs lost approximately 7–8% body weight compared to obese individuals receiving a low-calorie diet who lost approximately 3–7% body weight [24]. Moreover, intake of low-calorie diet plans has resulted in weight gain and deterioration in metabolic control within a year of post-intervention in obese T2D patients [14].

Adhering to lifestyle changes inclusive of diet and physical activity regime have been proven to be challenging for patients with T2D. Patients may find lifestyle behaviors (for example, diet and physical activity) hard to change and maintain for long periods [34]. A recent study in 2014 conducted in Nepal reported, 87.5% of T2D patients did not adhere to their dietary advice while 42.1% patients did not adhere to their exercise regime [35]. In another study in Kuwait, rates of nonadherence to dietary and exercise recommendations were estimated as 63.5% and 64.4%, respectively [34]. Evidences have shown that noncompliance to dietary and physical activity interventions in weight loss studies have resulted in an overall dropout rate between 20%-65% [36, 37]. Literature has highlighted that patient's compliance and subsequent glycemic control are markedly influenced by barriers related to dietary habits, lifestyle, and treatment regimens [37]. Furthermore, compliance to the daily intake of the MRs was essential for a successful weight loss program. Evidences reported a high attrition rate in MR trials due to mainly noncompliance to the dietary recommendations and dietary fatigue due to usage of same or similar MRs [39, 40]. Davis et al., (2010) reported an overall attrition rate of 43.2% in obese adults receiving MRs as a result of dietary fatigue using the same or similar MRs [39, 40]. Nonadherence to the MRs may influence weight regain to a certain extent during the weight maintenance phase [39]. Therefore, adherence towards the program recommendations is the cornerstone for successful weight loss interventions.

A number of different counseling interventions have been found to have an effect on weight loss [41, 42]. A meta-analysis of dietary counseling found that compared to usual care, there was a mean treatment effect of a 6% initial body weight reduction at one-year which was clinically significant [41]. Evidences from several studies showed that counseling technique have been successful in initiating weight loss behavior change in overweight/obese patients [43] and motivational interviewing (MI) counseling technique resulted in superior weight loss compared to standard weight loss counseling [43, 44]. A meta-analysis identified 11 randomized control trials that examined the effect of MI on weight loss. The authors found that MI significantly improved weight loss compared to controls as the MI group lost 1.47 kg more than the controls at six months [42]. In a further meta-analysis, it was found that MI had statistically significant positive impact on weight [45].

Motivational interviewing is a counseling technique that involves enhancing patient’s motivation to change [46, 47]. Motivational interviewing focuses on exploring and resolving ambivalence and centers on motivational processes within the individual that facilitate change [46, 47]. The method differs from more “coercive” or externally-driven methods for motivating change as it does not impose change (that may be inconsistent with the person's own values, beliefs or wishes), but, rather supports change in a manner congruent with the individual's own values and concerns [46]. Motivational interviewing uses guiding principles that emphasizes a collaborative therapeutic relationship in which the autonomy of the patient is respected [47]. Motivational interviewing was originally developed for treating substance abuse and other behavioral problems. Recent systematic reviews and meta-analyses offer evidence that MI is a promising technique to aid weight reduction [42] and reinforcing behavior change to weight loss [43, 44]. The use of MI among overweight/obese T2D patients had led to increased adherence to the weight loss program recommendations [48], enhanced weight loss [49] and sustained weight loss through an 18-month weight maintenance phase [49]. Motivational interviewing has also been incorporated in lifestyle intervention trials with MRs intake though the evidence is not abundant [17, 50]. Motivational interviewing could be a potentially effective method to increase compliance towards the MRs intake, and promote and sustain weight loss in overweight/obese T2D patients.

**2.1 Study Rationale**

The Malaysian DiabCare studies and local audits have shown that more than 70% of the Malaysian T2D patients had poor glycemic control (A1C >7%) [3-5], and obesity was prevalent in 83.4% of the T2D patients [7]. Several local surveys have reported that generally, Malaysian T2D patients ate higher energy (1600-1700 kcal/day), carbohydrate (55-60% of total energy) and fat (27-30% of total energy) [51-53], and had lower physical activity levels, where more than 50% of T2D patients were physical inactive [54, 55]. These studies indicated that the Malaysian T2D patients had difficulty adhering to lifestyle recommendations. Moreover, there have not been substantial studies evaluating the efficacy of structured lifestyle intervention with the incorporation of MRs and behavioral counseling such as the MI counseling in the local T2D patients. There was, however, a small study conducted for a period of three months, evaluating the efficacy of MNT with lifestyle recommendations in one-hundred and four local T2D patients [56]. In this study, the A1C reduced significantly with the intervention provided (7.6 ± 1.2 to 7.2 ± 1.1%, p<0.001), but there were no changes in the body weight (68.0 ± 12.5 to 67.6 ± 12.4 kg, p>0.05) [56]. In view of poor adherence to lifestyle recommendations and lack of large scale lifestyle-based intervention studies among the local T2D patients, the proposed study is justified to find out if structured lifestyle intervention with the incorporation of MRs and behavioral counseling such as the MI counseling would benefit the T2D patients. The novelty of this study is the use of structured lifestyle intervention with the tDNA care and the use of MI counseling to improve adherence to recommendations as delineated in the tDNA care.

**3.0 Methodology**

**3.1 Study Design**

This is a randomized open-label clinical trial which will be conducted in overweight/obese T2D patients. Patients will be randomized into three groups: tDNA care group receiving motivational interviewing counseling technique (tDNA-MI), similar tDNA intervention group receiving conventional counseling technique (tDNA-CC) and usual care (UC) group following standard diabetes care. This study will be conducted for a period of one-year consisting of 6 months of intervention phase and subsequent 6 months of follow up phase.

**3.2 Sample Size**

The sample size was calculated based on published data by Balalcazar et al. (2010) [57] in the Look AHEAD study results for A1C using intensive lifestyle vs. usual care. The differences between the A1C means calculated was 0.4 at 80% power with type 1 error of 5% (α =5%). The sample size obtained was 115 patients per arm inclusive of 15% dropout rate. Hence, the total sample size needed for this study is 230 patients (115 patients receiving usual care; 115 patients receiving tDNA care).

| Difference between means (A1C) | 0.4 |
| --- | --- |
| Common Standard Deviation | 1.0 |
| Power | 80% |
| Needed evaluable sample size | 115 per arm (with 15% drop-out) |

**Total sample size = 230 patients**

**3.3 Study Site**

Overweight/obese T2D patients will be recruited from primary care health clinic (Seremban).

**3.4 Inclusion &Exclusion Criteria**

**3.4.1 Inclusion Criteria**

Patients will be recruited if they are male or female, diagnosed with T2D and requiring weight loss, from all ethnicity, aged 30 to 65 years, has BMI of >23 kg/m² (Malaysian CPG cut-off point for overweight/obesity) and have A1C levels of 7-9%. Individuals confirmed with T2D treated with non-pharmacology therapy (diet and exercise) and with one or more oral hypoglycemic agents are eligible. Medications should be already optimized with no change in pharmacotherapy in the last 3 months. Patients who have seen the dietitian and have not achieved A1C reduction after 3 months are also eligible. Patient is also recruited if willing to comply with the study procedures

**3.4.2 Exclusion Criteria**

Individuals for whom weight loss might not be safe, those who may have difficulty adhering to the lifestyle intervention, or those with medical conditions that might interfere with intervention goals will be excluded. They include:

- Type 1 DM
- Type 2 DM on basal or multiple insulin injections
- Patients with weight loss exceeding 5 kg in the past three months
- Current use of medications/meal replacements for weight loss
- Chronic alcoholism
- History of bariatric surgery, small bowel resection, or extensive bowel resection
- Chronic treatment with systemic corticosteroids. Use of hormone replacement therapy or oral contraceptives will not lead to exclusion
- Currently pregnant or nursing
- Cancer requiring treatment for the past 5 years, except of non-melanoma skin cancers or cancers that have been clearly cured
- HIV- positive (self-report) due to effects or weight and body composition of HIV and medications treating HIV
- Cardiovascular disease (heart attack or procedure within past 3 months or participation in cardiac rehabilitation program within last 3 months, stroke or history/treatment for transient ischemic attacks in the past 3 months, or documented history of pulmonary embolus for the past 6 months)
- Renal disease - eGFR<60 ml/min (based MDRD) or currently receiving dialysis
- Patients who plan to relocate where it does not permit full participation in the study

**3.4.3 Randomization**

Patients will be randomized to receive tDNA care (intervention group) or usual care (control group). Patients in the tDNA care would be further randomized to receive motivational interviewing counseling or conventional counseling techniques. Randomization would be carried out using the computerized random generation of numbers by the random allocation software [58].

**3.4.4 Intervention**

*Structured lifestyle intervention.*

Patients in the tDNA intervention group will undergo an initial risk stratification based on the tDNA algorithm [23]. These patients will be prescribed with MNT consisting of a structured low*-*calorie meal plan (1200 kcal/day for female or 1500 kcal/day for male patients) using normal foods, incorporation of 1-2 servings of diabetes-specific formula as meal replacements according to the tDNA protocol, and a physical activity prescription of at least 150 minutes per week. Patients will receive education using the tDNA toolkit and consisting of a flip chart on healthy eating, 14-day meal plan, and culturally-adapted information on physical activity and exercise.

The tDNA-MI subgroup will receive counseling incorporating motivational interviewing principles [46,47]. The principles of motivational interviewing include expressing empathy through reflective listening, developing discrepancy between patients’ goals or values and their current behavior, avoiding argument and direct confrontation, adjusting to patient resistance and supporting self-efficacy and optimism [46, 47]. The tDNA-CC subgroup will receive conventional counseling focusing on empathetic listening, education, persuasion, and encouragement.

*Usual care*

The UC comparator group will follow the clinical care pathway of the Malaysian Clinical Practice Guidelines for Type 2 Diabetes Mellitus (2009) [59] and will receive advice to follow a conventional low-calorie diet plan (1200 kcal/day for female or 1500 kcal/day for male patients) using normal foods with standard diabetes support and lifestyle education. Dietary counseling will be conducted by the dietitian based on individualized care using conventional counseling technique to facilitate positive behavioral change toward weight loss.

*Frequency of contact*

Patients in the tDNA-MI and tDNA-CC will be followed up monthly by the dietitian and physician during the intervention phase and subsequently every 3 months during the follow-up phase. Patients in the UC group will be followed-up 3 monthly throughout the study duration by the dietitian and physician. All patients were required to keep a 3-day food record and one week of exercise diary.

*Follow-up phase*

All patients will be followed up for six months’ post intervention. The follow up phase would involve mainly observation of patients’ adherence to the previously provided intervention.

**3.4.5 Study Run-In**

Eligible patients will complete a two-week run-in period prior to intervention. They will be asked to record information about diet and physical activity daily during this period. Successful completion of self-monitoring will be required for further eligibility.

**3.4.6 Withdrawal criteria**

Patients receiving the tDNA care who failed to attend the monthly interval follow-up >3 consecutive times will be dropped from study. Patients with consistent compliance of <50% towards the diabetes-specific formula for more than three consecutive times and voluntarily wants to quit the study will also be dropped out. Furthermore, patients who failed to adhere to the study protocol will be discontinued from the study.

Similarly, patients in the UC group who do not keep their appointments at 3 and 6 months would be dropped from the study.

**3.5 Evaluation Parameters**

**Socio-demography:** Socio-demography data including age, marital status, education and income level, medical history, medication intake would be collected via a questionnaire and thereafter patients would be screened for eligibility criteria.

**Weight and body composition:** Weight and height will be measured using weighing scale and stadiometer and body mass index will be calculated. Waist-circumference will be taken at the midpoint between the lower margin of the last palpable rib and the top of the iliac crest with a measuring tape. Percentage body fat will be measured using the Bioelectrical-Impedance Analysis (BIA).

**Metabolic outcomes:** Blood will be withdrawn from patients after an overnight fast of 10-12 hours. A1C, fasting plasma glucose, lipid profile, and inflammatory marker (hsCRP) will be analyzed using automated procedures. Seated blood pressure will be measured twice, using an automated device after a 5-minute rest. Prior to measurement, patients’ blood pressure will be measured in both arms to determine which arm should be used for future measurements. The arm that gives higher systolic reading will be used for all future testing.

**Dietary intake and adherence:** Patients will be required to keep a 3-day food record (2 weekdays and 1 weekend, consecutively) at monthly basis for the tDNA care group and every 3 monthly bases for UC group. Food portion intake will be recorded using household measurements. Colored food albums with photographs of actual serving sizes will be used to quantify the food portions consumed. Written instructions with example of a one-day food record will be given to all patients. The nutrient content of each food was analyzed using Nutritionist Pro computer software (V5.1.0, Axxya Systems, Texas USA), primarily based on the nutrient composition of Malaysian foods [60].

Adherence to the meal replacement provided will be recorded in a form on daily basis and empty sachets will be re-collected.

**Physical activity record:** Type, frequency and duration of exercise engaged by the patients will be recorded at daily basis in an exercise diary. Patients’ physical activity level would be measured using the International Physical Activity Questionnaire (IPAQ)-Short Form validated for Malaysians [61].

**Eating self-efficacy:** Patients self-efficacy level following a weight loss program will be assessed using the Weight Efficacy Lifestyle (WEL) questionnaire. It is a commonly used measure of eating self-efficacy consisting of 20-items and five situational factors which includes negative emotions, availability, social pressure, physical discomfort, and positive activities [62]. Patients are to rate in a scale from 0 (not confident) to 10 (very confident) on whether they can maintain a healthy diet in those tempting situations. Higher the WEL scores higher the self-efficacy to resist eating.

**3.6 Statistical Analysis**

Statistical analysis will be done using the statistical package for social sciences (SPSS) version 22 software (SPSS Inc., Chicago, IL, US). Data will be checked for normality. Descriptive statistics (mean, SD, median, IQR, SE, frequency, and percentage of patients) will be used to describe the baseline data. The parametric data will be presented as mean ± SD where comparisons between groups will be done using one-way analysis of variance (ANOVA) statistical analysis for continuous variables. Kruskal-Wallis test will be used for comparison between groups for non-parametric data presented as median ± IQR. The χ² tests will be used for comparison between groups for categorical data.

Absolute change and percentage change in outcomes will be computed from baseline to 6 or 12 months of the intervention period. General linear model will be used for comparisons of changes in outcomes within groups. A p-value of less than 0.05 will be as considered statistically significant.

Figure 1: Transcultural Diabetes-Specific Nutrition Algorithm (tDNA)


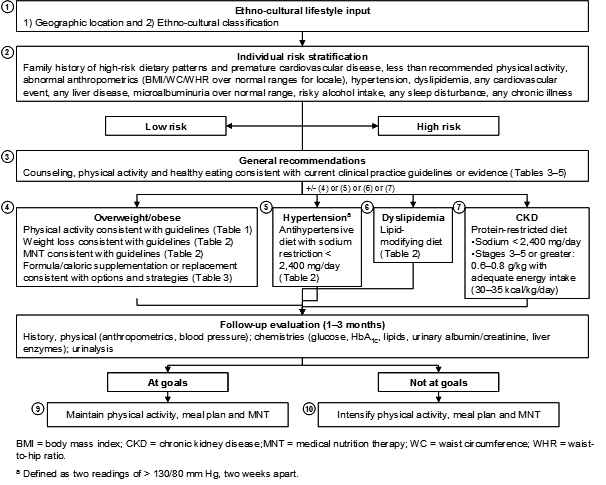


Ref: Zanariah H, WSS Chee, YC Chia, et al. 2013. Transcultural Diabetes-Specific Nutrition Therapy Algorithm: Malaysian Application. Int J of Endocrinology (accepted 10 Oct 2013*)*

**Figure 2: Treatment Algorithm for Management of Type 2 Diabetes Mellitus**

**
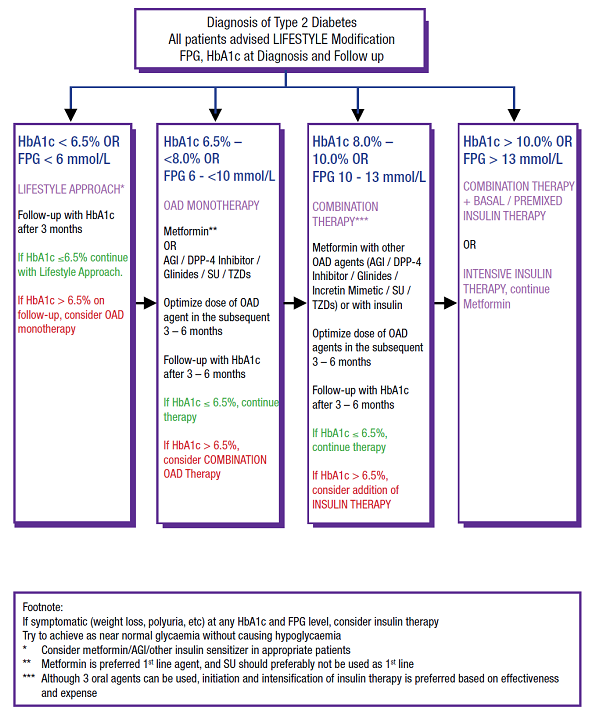
**

Ref: Clinical Practice Guidelines for T2D (MOH, 2012)

**4.0 References**

[1] Institute for Public Health. National Health and Morbidity Survey 2015 (NHMS 2015). Vol. II: Non-Communicable Diseases, Risk Factors & Other Health Problems. Malaysia Institute for Public Health, Ministry of Health: Kuala Lumpur, Malaysia; 2015

[2] Letchuman GR, Wan Nazaimoon WM, Wan Mohamad WB, Chandran LR, Tee GH, Jamaiyah H, Isa MR, Zanariah H, Fatanah I, Ahmad Faudzi Y. Prevalence of Diabetes in the Malaysian National Health Morbidity Survey III 2006. The Malaysian National Health Morbidity Survey III 2006. Med J Malaysia. 2010; **65(3)**:173-179

[3] Mafauzy M, Hussein Z, Chan SP. The status of diabetes control in Malaysia: results of DiabCare 2008. Med J Malaysia. 2011; **66(3)**:175-181

[4] Mohamed M, Diabcare-Asia 2003 Study Group. An audit on diabetes management in Asian patients treated by specialists: the DiabCare-Asia 1998 and 2003 studies. Curr Med Res Opin. 2008; **24(2)**:507-514

[5] Mafauzy M, Zanariah H, Avideh N, Chan SP. DiabCare 2013: a cross-sectional study of hospital based diabetes care delivery and prevention of diabetes related complications in Malaysia. Med J Malaysia. 2016: **71(4)**: 177-185

[6] Institute for Public Health. The Third National Health and Morbidity Survey (NHMS III) 2006. Nutritional Status. Malaysia Institute for Public Health, Ministry of Health: Kuala Lumpur, Malaysia; 2008

[7] Feisul MI, Azmi S, editors. National Diabetes Registry Report, Volume 1, 2009-2012. 1st ed. Kuala Lumpur, Ministry of Health Malaysia; 2013

[8] Zaki M, Robaayah Z, Chan SP, Vadivale M, Lim TO. Malaysia Shape of the Nation (MySoN): a primary care based study of abdominal obesity in Malaysia. Med J Malaysia. 2010; **65(Suppl A)**:143-149

[9] Sung K-C, Jeong W-S, Wild SH, Byrne CD. Combined Influence of Insulin Resistance, Overweight/Obesity, and Fatty Liver as Risk Factors for Type 2 Diabetes. Diabetes Care. 2012; **35(4)**:717-722

[10] Guilherme A, Virbasius JV, Puri V, Czech MP. Adipocyte dysfunctions linking obesity to insulin resistance and type 2 diabetes. Nat Rev Mol Cell Biol. 2008; **9(5)**:367–377

[11] Anderson JW, Kendall CWC, Jenkins DJA. Importance of weight management in type 2 diabetes: Review with meta-analysis of clinical studies. J Am Coll Nutr. 2003; **22(5)**:331-339

[12] Williamson DF, Thompson TJ, Thun M, Flanders D, Pamuk E, Byers T. Intentional weight loss and mortality amongst overweight individuals with diabetes. Diabetes Care. 2000; **23**:1499-1504

[13] Wing RR. Use of very-low-calorie diets in the treatment of obese persons with non-insulin-dependent diabetes mellitus. J Am Diet Assoc. 1995; **95(5)**:569-729

[14] Wadden TA. Treatment of obesity by moderate and severe caloric restriction: results of clinical research trials. Ann Intern Med. 1993; **119(7)**:688-693

[15] Wallman K, Plant LA, Rakimov B, Maiorana AJ. The effects of two modest of exercise on aerobic fitness and fat mass in an overweight population. Res Sports Med. 2009; **17**:156-170

[16] Carels RA, Darby LA, Rydin S, Douglass OM, Cacciapaglia HM, O’Brien WH. The relationship between self-monitoring, outcome expectancies, difficulties with eating and exercise and physical activity and weight loss treatment outcomes. Ann Behav Med. 2005; **30(3)**:182-190

[17] The Look AHEAD Research Group. Reduction in weight and cardiovascular disease risk factors in individuals with type 2 diabetes. One-year results of the Look AHEAD trial. Diabetes Care. 2007; **30**:1374-1383

[18] Wing RR. Behavioural treatment of obesity. Diabetes Care. 1993; **16**:193-199

[19] Wing RR, Anglin K. Effectiveness of a behavioural weight control program for blacks and whites with NIDDM. Diabetes Care. 1996; **19**:409-413

[20] Tuomilehto J, Lindstrom J, Eriksson JG, Valle TT, Hämäläinen H, Ilanne-Parikka P, Keinänen-Kiukaanniemi S, Laakso M, Louheranta A, Rastas M, Salminen V, Aunola S, Cepaitis Z, Moltchanov V, Hakumäki M, Mannelin M, Martikkala V, Sundvall J, Uusitupa M for the Finnish Diabetes Prevention Study Group. Prevention of Type 2 Diabetes Mellitus by Changes in Lifestyle amongst Subjects with Impaired Glucose Tolerance. N Engl J Med. 2001; **344**:1343-1350

[21] Chen L, Pei J-H, Kuang J, Chen H-M, Chen Z, Li Z-W, Yang H-Z. Effect of lifestyle intervention in patients with type 2 diabetes: A meta-analysis. Metab Clin Exp. 2015; **64(2)**:338-347

[22] Mechanick JI, Marchetti AE, Apovian C, Benchimol AK, Bisschop PH, Bolio-Galvis A, Hegazi RA, Jenkins D, Mendoza E, Sanz ML, Sheu WH, Tatti P, Tsang MW, Hamdy O. Diabetes-specific nutrition algorithm: a transcultural program to optimize diabetes and prediabetes care. Curr Diab Rep. 2012; **12(2)**:180-194

[23] Hussein Z, Hamdy O, Chia YC, Lim SL, Natkunam SK, Hussain H, Tan MY, Sulaiman R, Nisak B, Chee WSS, Marchetti A, Hegazi RA, Mechanick JI. Transcultural Diabetes Nutrition Algorithm : A Malaysian Application. Int J Endocrinol. 2013; **(Article ID 679396)**:7pages

[24] Heymsfield SB, van Mierlo CAJ, van der Knaap HCM, Heo M, Frie HI. Weight management using a meal replacement strategy: meta and pooling analysis from six studies. Int J Obes. 2003; **27**:537–549

[25] Noakes M, Foster PR, Keogh JB, Clifton PM. Meal replacements are as effective as structured weight-loss diets for treating obesity in adults with features of metabolic syndrome. J Nutr. 2004; **134(8)**:1894-1899

[26] Treyzon L, Chen S, Hong K, Yan E, Carpenter CL, Thames G, Bowerman S, Wang H-J, Elashoff R, Li Z. A controlled trial of protein enrichment of meal replacements for weight reduction with retention of lean body mass. Nutr J. 2008; **7**:23

[27] Tatti P, di Mauro P, Neri M, Pipicelli, Mussad VA. Effect of a low-calorie high nutritional value formula on weight loss in type 2 diabetes mellitus. Mediterr J Nutr Metab. 2010; **3**:65-69

[28] Ashley JM, Herzog H, Clodfelter S, Bovee V, Schrage J, Pritsos C. Nutrient adequacy during weight loss interventions: a randomized study in women comparing the dietary intake in a meal replacement group with a traditional food group. Nutr J. 2007; **6**:12

[29] Wadden TA, West DS, Neiberg RH, Wing RR, Ryan DH, Johnson KC, Foreyt JP, Hill JO, Trence DL, Vitolins MZ, Look AHEAD Research Group. One-year weight losses in the Look AHEAD study: factors associated with success. Obesity (Silver Spring). 2009; **17(4)**:713-712

[30] Rothacker QD. Five-year self-management of weight using meal replacements: comparison with matched controls in rural Wisconsin. Nutrition. 2000; **16(5)**:344-348

[31] Cheskin LJ, Mitchell AM, Jhaveri AD, Mitola AH, Davis LM, Lewis RA, Yep MA, Lycan TW. Efficacy of meal replacements versus a standard food-based diet for weight loss in type 2 diabetes: a controlled clinical trial. Diabetes Educ. 2008; **34(1)**:118-127

[32] Li Z, Hong K, Saltsman P, DeShields S, Bellman M, Thames G, Liu Y, Wang HJ, Elashoff R, Heber D. Long-term efficacy of soy-based meal replacements vs. an individualized diet plan in obese type II DM patients: relative effects on weight loss, metabolic parameters, and C-reactive protein. Eur J Clin Nutr. 2005; **59(3)**:411-418

[33] Yip I, Go VL, DeShields S, Saltsman P, Bellman M, Thames G, Murray S, Wang HJ, Elashoff R, Heber D. Liquid meal replacements and glycaemic control in obese type 2 diabetes patients. Obes Res. 2001; **9(Suppl 4)**:341S-347S

[34] Serour M, Alqhenaei H, Al-Saqabi S, Mustafa A, Ben-Nakhi A. Cultural factors and patients adherence to lifestyle measures. Br J Gen Pract. 2007; **57**:291-295

[35] Parajuli J, Saleh F, Thapa N, Ali. Factors associated with nonadherence to diet and physical activity amongst nepalese type 2 diabetes patients; a cross sectional study. BMC Research Notes. 2014; **7**:758

[36] Foster G, Wyatt HR, Hill JO, McGuckin BG, Brill C, Mohammed BS, Szapary PO, Rader DJ, Edman JS, Klein S. A Randomized Trial of a Low-Carbohydrate Diet for Obesity. N Engl J Med. 2003; **348**:2082-2090

[37] Wu T, Gao X, Chen M, Van Dam RM. Long-term effectiveness of diet-plus-exercise interventions vs. diet-only interventions for weight loss: a meta-analysis. Obes Rev. 2009; **10**:313-323

[38] O'Rahilly S, Barroso I, Wareham NJ. Genetic factors in type 2 diabetes: the end of the beginning? Science. 2005; **307(5708)**:370-373

[39] Davis LM, Coleman C, Kiel J, Rampolla J, Hutchisen T, Ford L, Andersen WS, Hanlon-Mitola A. Efficacy of a meal replacement diet plan compared to a food-based diet plan after a period of weight loss and weight maintenance: a randomized controlled trial. Nutr J. 2010; **9**:11

[40] Crichton GE, Howe PRC, Buckley JD, Coates AM, Murphy KJ, Bryan J. Long-term dietary intervention trials: critical issues and challenges. Trials. 2012; **13**:111

[41] Dansinger ML, Tatsioni A, Wong JB, Chung M, Balk EM. Meta-analysis: the effect of dietary counseling for weight loss. Ann Intern Med. 2007; **147(1)**:41-50

[42] Armstrong MJ, Mottershead TA, Ronksley PE, Sigal RJ, Campbell TS, Hemmelgarn BR. Motivational interviewing to improve weight loss in overweight and/or obese patients: a systematic review and meta-analysis of randomized controlled trials. Obes Rev. 2011; **4**:709-723

[43] Rose SA, Poynter PS, Anderson JW, Noar SM and Conigliaro J. Physician weight loss advice and patient weight loss behavior change: a literature review and meta-analysis of survey data. Int J Obes. 2013; **37**:118-128

[44] Pollak KI, Alexander SC, Coffman CJ, Tulsky JA, Lyna P, Dolor RJ, James IE, Brouwer RJ, Manusov JR, Østbye T. Physician communication techniques and weight loss in adults: project CHAT. Am J Prev Med. 2010; **39(4)**:321-328

[45] Lundahl B, Moleni T, Burke BL, Butters R, Tollefson D, Butler C, Rollnick S. Motivational interviewing in medical care settings: a systematic review and meta-analysis of randomized controlled trials. Patient Educ Couns. 2013; **93(2)**:157-168

[46] Rollnick S, Miller WR. What is motivational interviewing? Behav Cogn Psychother. 1995; **23**:325-334

[47] Miller WR, Rollnick S. Motivational interviewing: Helping people change. New York: Guilford Press; 2012

[48] Smith DE, Heckemeyer CM, Kratt PP, Mason DA. Motivational interviewing to improve adherence to a behavioral weight control program for older obese women with NIDDM. A pilot study. Diabetes Care. 1997; **20**:52-54

[49] West DS, DiLillo V, Bursac Z, Gore SA, Greene PG. Motivational interviewing improves weight loss in women with type 2 diabetes. Diabetes Care. 2007; **30**:1081-1087

[50] Rohrer JE, Takahashi P. Should overweight and obese primary care patients be offered a meal replacement diet? Obes Res Clin Pract. 2008; **2(4)**:I-II

[51] Koo HC, Sathyasurya DR, Hazizi AD, Hamid Jan JM. Effect of ethnicity, dietary intake and physical activity on plasma adiponectin concentrations amongst Malaysian patients with type 2 diabetes mellitus. Int J Endocrinol Metab. 2013; **11**:167-174

[52] Firouzi S, Barakatun-Nisak MY, Nor Azmi K. Nutritional status, glycemic control and its associated risk factors among a sample of type 2 diabetic individuals, a pilot study. J Res Med Sci. 2015; **20(1)**:40-46

[53] Tan MC, Ng OC, Wong TW, Joseph A, Abdul Rahman H, Abdul Aziz R. Dietary compliance, dietary supplementation and traditional remedy usage of type 2 diabetic patients with and without cardiovascular disease. Clin Nutr Res. 2015; **4(1)**:18-31

[54] Tan MY, Magarey J. Self-care practices of Malaysian adults with diabetes and sub-optimal glycaemic control. Patient Educ Couns. 2008; **72**:252-267

[55] Siti Khuzaimah AS, Habibah Humairah Z, Zaliha R, Nur Aswadani B. Self-care practices of diabetes patients among three ethnics in Malaysia: Malay, Chinese and Indian. Proceedings of International Conference on Public Policy and Social Sciences, UITM Melaka, Malaysia; 2012

[56] Barakatun Nisak MY, Ruzita AT, Norimah AK, Kamaruddin NA. Medical nutrition therapy administered by a dietitian yields favourable diabetes outcomes in individual with type 2 diabetes mellitus. Med J Malaysia. 2013; **68(1)**:18-23

[57] Belalcazar LM, Reboussin DM, Haffner SM, Hoogeveen RC, Kriska AM, Schwenke DC, Tracy RP, Pi-Sunyer FX, Ballantyne CM for the Look AHEAD Research Group. A 1-Year Lifestyle Intervention for Weight Loss in Individuals with Type 2 Diabetes Reduces High C-Reactive Protein Levels and Identifies Metabolic Predictors of Change. Diab Care. 2010; **33**:2297–2303

[58] Saghaei M. Random allocation software for parallel group randomized trials. BMC Med Res Methodol. 2004; **4**:26 Available from: http://www.pubmedcentral.nih.gov/articlerender.fcgi?artid=533876&tool=pmcentrez&rendertype=abstract

[59] Ministry of Health Malaysia. Clinical Practice Guidelines for the Management of Type 2 Diabetes Mellitus. 4th ed. Kuala Lumpur: Ministry of Health; 2009. Available from: http://www.acadmed.org.my/

[60] Tee ES, Noor MI, Azudin MN, Idris K. Nutrient Composition of Malaysian Foods, 4th ed. Ministry of Health Malaysia; 1997

[61] Shamsuddin N, Koon PB, Zulkifli S, Zakaria S, Noor MI. Reliability and Validity of Malay Language Version of International Physical Activity Questionnaire ( IPAQ-M ) amongst the Malaysian Cohort Participants. Int J Public Health Res. 2015; **5(2)**:643-653

[62] Clark MM, Abrams DB, Niaura RS, Eaton CA, Rossi JS. Self-efficacy in weight

management. J Consult Clin Psychol. 1991;59:739-744.
